# Design and Development of a Run-Time Monitor for Multi-Core Architectures in Cloud Computing

**DOI:** 10.3390/s110403595

**Published:** 2011-03-25

**Authors:** Mikyung Kang, Dong-In Kang, Stephen P. Crago, Gyung-Leen Park, Junghoon Lee

**Affiliations:** 1 Information Sciences Institute (ISI), University of Southern California (USC), Arlington, VA 22203, USA; E-Mails: mkkang@isi.edu (M.K.); dkang@isi.edu (D.-I.K.); crago@isi.edu (S.P.C.); 2 Department of Computer Science and Statistics, Jeju National University, Jeju 690-756, Korea; E-Mail: glpark@jejunu.ac.kr

**Keywords:** Run-Time Monitor, multi-core architectures, cloud computing, QoS, library instrumentation, performance counter

## Abstract

Cloud computing is a new information technology trend that moves computing and data away from desktops and portable PCs into large data centers. The basic principle of cloud computing is to deliver applications as services over the Internet as well as infrastructure. A cloud is a type of parallel and distributed system consisting of a collection of inter-connected and virtualized computers that are dynamically provisioned and presented as one or more unified computing resources. The large-scale distributed applications on a cloud require adaptive service-based software, which has the capability of monitoring system status changes, analyzing the monitored information, and adapting its service configuration while considering tradeoffs among multiple QoS features simultaneously. In this paper, we design and develop a Run-Time Monitor (RTM) which is a system software to monitor the application behavior at run-time, analyze the collected information, and optimize cloud computing resources for multi-core architectures. RTM monitors application software through library instrumentation as well as underlying hardware through a performance counter optimizing its computing configuration based on the analyzed data.

## 1. Introduction

Cloud computing is a new information technology trend that moves computing and data away from desktops and portable PCs into large data centers. The basic principle of cloud computing is to deliver applications as services over the Internet as well as infrastructure. A cloud is the type of a parallel and distributed system consisting of a collection of inter-connected and virtualized computers that are dynamically provisioned and presented as one or more unified computing resources [1]. The large-scale distributed applications on a cloud require adaptive service-based software, which has the capability of monitoring the system status changes, analyzing the monitored information, and adapting its service configuration while considering tradeoffs among multiple QoS features simultaneously.

The cloud provides scalable processing power and several kinds of connectable services. This distributed architecture has many similarities with a typical wireless sensor network, where a lot of motes, which are responsible for sensing and local preprocessing, are interconnected with wireless connections [2]. While wireless sensor networks are limited in their processing power, battery life and communication speed, cloud computing usually offers the opposite features, which makes it attractive for long term observations, analysis and use in different kinds of environments and projects. These sensing data could be stored in one or more cloud computing facilities, and could be supplemented in operation on by extensive sensor data, collected and made available in near real-time.

Recently, multi-core and many-core architectures are becoming more and more popular due to the diminishing returns from traditional hardware innovations such as caching and deep pipeline architectures. With more cores in a processor, it is easier to get performance gains over traditional approaches by parallelizing applications. In addition, traditional processors consume large amounts of power to achieve high performance by using high frequencies. By using multiple cores at a lower frequency, and consequently lower voltage, multi-core architectures can achieve higher performance with lower power consumption.

There have been many multi-core processors from commercial vendors [3–5]. Among them, Tilera Corporation offers three processor families with the largest number of cores on a general-purpose chip available on the market [6]. Boeing has developed a processor called MAESTRO, to be used in space, based on the first Tilera processor, TILE64 [6,7]. The TILE64 has 64 cores on a chip. Each core has a three-instruction-wide Very Long Instruction Word (VLIW) pipeline, memory management unit, L1 and L2 cache, so each core itself is a complete processor, which can run a complete operating system like Linux (although more commonly, a single operating system instantiation is used to control multiple cores). The cores are interconnected with mesh networks, the processor executes at up to 866 MHz to achieve up to 443 billion operations per second. The Maestro chip is similar to the TILE64 except that Maestro has a floating point unit in each core, radiation-hardening by design, 49 cores instead of 64, and runs at a lower frequency.

We aimed to design a Run-Time Monitor (RTM) which is a system software to monitor the characteristics of applications at run-time, analyze the collected information, and optimize resources on a cloud node which consists of multi-core processors. The rest of the paper is organized as follows. In Section 2, the system architecture is briefly described. Our proposed Run-Time Monitor is described in Section 3. Implementation results are described in Section 4, and Section 5 concludes the paper.

## 2. System Architecture

### 2.1. Eucalyptus and OpenStack

**Figure 1.**
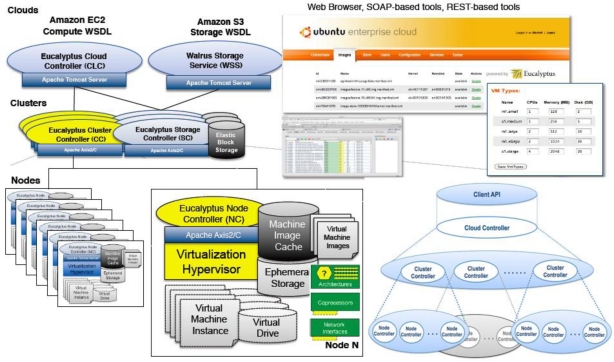
The resource topology structure of Ecalyptus.

The Elastic Utility Computing Architecture for Linking Your Programs To Useful Systems (Eucalyptus) project began at the University of California at Santa Barbara, and was mainly targeted at building a private open-source cloud platform [8]. Currently Eucalyptus is an open-source implementation of Amazon Elastic Compute Cloud (EC2) and compatible with most business interfaces [9,10]. Eucalyptus is an elastic computing structure that can be used to connect the user’s programmers to the useful systems and it is an open-source infrastructure using clusters or workstations implementation of elastic, utility, and cloud computing. Figure 1 demonstrates the topology structure of Eucalyptus resources.

In this figure, the node controller is a component running on the physical resources. On each node, all kinds of virtual machine entities can run. Logically connected nodes form a virtual cluster, and all nodes belonging to the same virtual cluster receive a command from the cluster controller and then report to the same controller. Parallel HPC applications often need to distribute large amounts of data to all compute nodes before or during a run [11]. In a cloud, these data are typically stored in a separate storage service. Distributing data from the storage service to all compute nodes is essentially a multicast operation.

**Figure 2.**
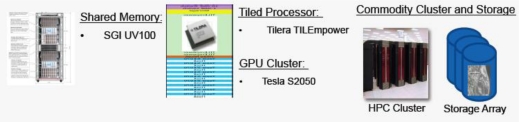
Heterogeneous processing test-beds.

Eucalyptus clouds can be run on heterogeneous machine types, that is, shared memory machines, tiled processor machines, and co-processors, as shown in Figure 2. Through HPC and Networking extensions, bandwidth reservation, node locality, switch topology, or private network interfaces can be considered on the clouds. University of Southern California (USC)/Information Sciences Institute (ISI) has been working on Dynamic On-Demand Computing System (DODCS) which is a heterogeneous high performance computing extension for Eucalyptus clouds. Since 2011, our DODCS team shifted the open source platform from Eucalyptus to OpenStack and we are now working on the OpenStack platform.

OpenStack is a collection of open source technologies delivering a massively scalable cloud operating system [12]. OpenStack is currently developing two interrelated projects: OpenStack Compute and OpenStack Object Storage. OpenStack Compute is software to provision and manage large groups of virtual private servers, and OpenStack Object Storage is software for creating redundant, scalable object storage using clusters of commodity servers to store terabytes or even petabytes of data. This paper targets multi-core processor with a single compute node for 10 multi-core boards. After receiving data and commands, each node processes data while monitoring performance and optimizing resources and then it returns the results to the cluster node.

On DODCS 3D heterogeneous processing test-beds, it will measure system responsiveness to analyst- and event-driven workloads, deploy heterogeneous processing test-bed for GED researchers, and support 3D “voxel” processing application development. In advance of implementation on these heterogeneous processing test-beds, our RTM is targeted at the basic tiled processors, TILE64 or TILEPro64, for processing parallel programs.

### 2.2. TILE64/TILEPro64

**Figure 3.**
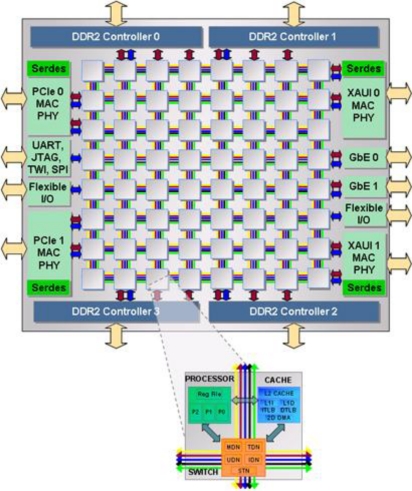
TILE64 block diagram [7].

TILE64 is the first commercial processor from Tilera Cooperation [7]. A block diagram of the processor is shown in Figure 3. The processor has 64 cores with an 8 by 8 array layout. Each core has a three-instruction-wide VLIW pipeline and an 8 KB L1 instruction cache, 8 KB data cache, and 64 KB L2 cache. The L2 cache is a unified 2-way cache. Each core is independent and is capable of executing its own operating system (or can be treated as part of a multiprocessor with a single operating system). The complete processor of 64 cores is cache coherent, using neighborhood caching.

The cores are interconnected with five networks: two networks are hardware controlled and are dedicated to memory and cache communication, one network is dedicated to I/O and the two remaining networks are dedicated to application communication. Having the five networks functionally distributed eliminates bottlenecks due to resource sharing as seen on single bus architectures. Each network is a two-dimensional mesh network with 32-bit width. Our implementation uses one of the two networks dedicated to the applications: the user dynamic network (UDN). The UDN network provides a very low latency and high bandwidth up to 31 Tbps, which provides a much better performance than traditional shared memory.

There are several I/O interfaces on the chip that eliminate external I/O circuits such as DDR2 memory controllers, PCIe, GbE, and XAUI that allow simpler design and smaller PCB space for a system. Another advantage of the processor is its low power consumption. For example, at 700 MHz, power per core is approximately 1/5 Watts. So, the total power for 64 cores is about 12 Watts, and with additional power consumption coming from memory controllers and I/O.

TILEPro64 is Tilera’s latest generation processor. It features 64 identical processor cores (tiles) interconnected with Tilera’s iMesh™ on-chip network [7]. Each tile is a complete full-featured processor, including integrated L1 and L2 cache and a non-blocking switch that connects the tile into the mesh. The TILEPro™ family incorporates Tilera’s Dynamic Distributed Cache (DDC™) technology that accelerates coherent cache performance by a factor of two, compared with other multicores.

## 3. Run-Time Monitor (RTM)

### 3.1. RTM Overview

**Figure 4.**
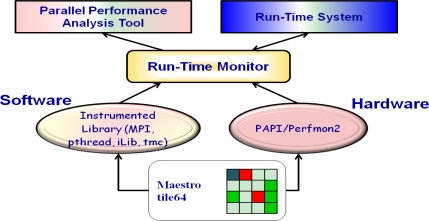
Run-Time Monitor Overview.

Run-Time Monitor is a system software to monitor the characteristics of applications at run-time. As shown in Figure 4, RTM monitors both application software and hardware. We implemented RTM through library instrumentation for software information and through Perfmon2/PAPI [18,19] for hardware information. The collected software and hardware information can be used by Parallel Performance Analysis Tool and the Run-Time system.

Performance of a parallel program is usually limited by the following reasons: (a) imbalance of computations among the threads/processes; (b) serialization due to synchronization; (c) waiting time due to synchronization; (d) contention of accessing shared memory. The information we collect for each API is as follows:

Timestamps: these time stamps will be used by RTM to construct the time sequence of the events and extract further information such as blocking time at the lock, critical section time, *etc.*

Event identifier: the event identifier is used to identify the type of the event, for example, pthread_mutex_lock(), pthread_mutex_unlock(), pthread_create(), pthread_join(), pthread_cond_ wait(), *etc.*

Data structure associated with the event: for each event, information is collected. Process id and thread id are collected for all events. For each event, the information is customized. For example, we collect the address of pthread mutex variable for pthread_mutex_lock(), pthread_unlock(), and pthread_cond_wait().

PC value of the calling function: identifying the location of an event in the source code is useful for debugging and performance optimization at the source code.

Affinity of the thread (on which CPU the thread runs): affinity denotes the CPU id where the thread runs. Since the Linux SMP scheduler schedules threads onto many cores dynamically, a thread can be scheduled onto multiple cores in its lifetime.

We are to monitor synchronization among the parallel threads/processes, blocking time due to synchronization, processing time within lock/unlock pairs, waiting time at barriers, and DMA activity if available. Monitoring of these factors can be used to detect performance bottlenecks of the application. Instrumenting pthread library can collect useful information to deduce the behavior of the parallel programs. We considered two techniques to instrument pthread libraries: a weak symbol binding technique or a library interposition technique. Each has its own pros and cons. A weak symbol binding technique needs source code changes of the pthread library, while library interposition doesn’t need those changes. Library interposition techniques only work when dynamic linking is used, while the weak symbol binding technique can be used for both dynamic linking and static linking.

### 3.2. RTM Libraries

RTM implementation targets multi-core processors such as Tilera’s TILE64 processor, the TILEPro64 processor or the MAESTRO processor. The implementation has been developed on top of a modified version of libraries such as iLib [7], MPI [13], pthread, and so on.

The TILE Processor is a new class of multi-core processing engine that delivers unprecedented levels of performance, flexibility and power efficiency. The device is fully programmable via a standard ANSI C environment, which provides an easy platform to port applications. The device implements Tilera’s SmartMesh Multi-core technology, which allows the ported applications to scale in multiple dimensions. The combination of multiple, C programmable processor cores and the mesh interconnect enables the device to achieve the performance of an ASIC in a software programmable solution, providing reduced development time and faster time-to-market. Tilera’s iLib API allows programmers to effectively utilize the TILE Architecture’s resources from C programs [7]. iLib applications are implemented as a set of processes, one per tile. These processes can communicate by streaming data over channels, passing data buffers via messages, or through shared memory.

Our implementation of MPI is based on a modified iLib implementation [14]. Since there are a few features needed by MPI that are not in iLib, such as non-blocking functions and support of MPI_ANY_TAG and MPI_ANY_SOURCE, we augmented the original iLib library provided by Tilera. Then, on top of the modified library, we implemented the full MPI library, following the MPI 1.2 standard. We tested our MPI library with several MPI test suites including an IBM test suite [15], Intel test suite [16], MPICH test suite [17], and SpecMPI test suite [18]. It passed all C-language tests in the suites except when the data size is too big for the memory on the TILExpress-64 evaluation board. In these cases, we reduced data sizes, and the tests passed.

### 3.3. Library Interposition

**Figure 5.**
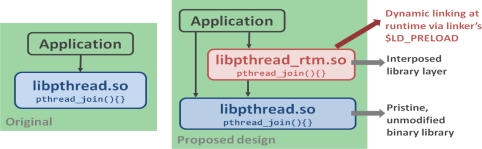
Library Interposition.

For the dynamic linking at runtime via preload, we exploited library interposition with the library instrumentation as shown in Figure 5.

Interposition is a technique that allows an additional function to be automatically called whenever a library function is called. In RTM model, an interposed library layer was added so that original library modification/recompilation is not needed, no source is needed for anything, and no recompilation/redo on library version update (only on API change), so in each interposed library, needed information was collected after calling the unmodified binary library.

**Figure 6.**
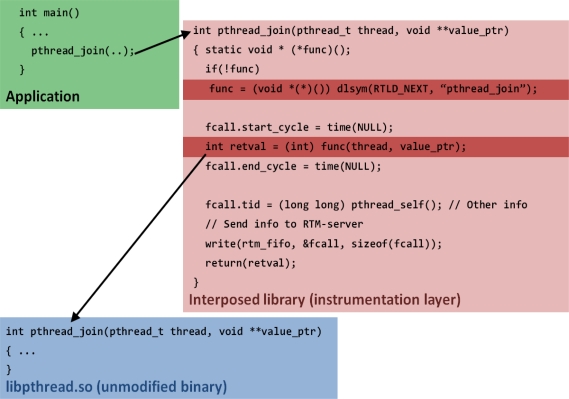
Sample Interposed Call.

Let’s see this sample interposed call, *pthread_join*, in Figure 6. In the application, pthread_join is called. Then, in the interposed library layer, the unmodified original library is called and the return value is saved. The *dlsym* is a routine that gives the user direct access to the dynamic linking facilities. The *dlsym* allows a process to obtain the address of a symbol defined within a shared object previously opened by *dlopen*. If handle is RTLD_NEXT, the search begins with the next object after the object from which *dlsym* was invoked. The *pthread_join* is the symbol’s name as a character string. And then other information needed for RTM was calculated, set, and saved in FIFO system. After saving the necessary information, the interposed library sends the information to the RTM server at the instrumentation layer. Basic library functions and inline/macro functions were instrumented for the interposition.

### 3.4. RTM Server and Client Model

**Figure 7.**
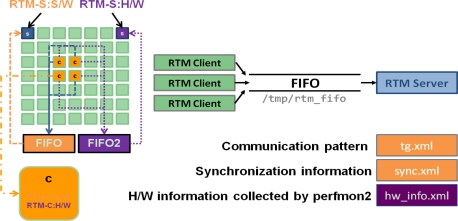
RTM Server and Client.

The RTM server and client concept is illustrated in Figure 7. After loading the RTM software and hardware servers, the RTM client program begins to run. Whenever an event happens on each tile, the RTM client sends the information to the RTM server using the system FIFO. Then the RTM software server calculates the communication pattern and synchronization information providing task graph and synchronization graph XML files periodically.

The RTM hardware server also collects hardware information from hardware clients on each tile through Perfmon2/PAPI. Perfmon2 is the hardware-based performance monitoring interface for Linux [19]. Performance Application Programming Interface (PAPI) aims to provide the tool designer and application engineer with a consistent interface and methodology for use of the performance counter hardware found in most major microprocessors [20]. PAPI enables software engineers to see, in near real time, the relation between software performance and processor events.

**Figure 8.**
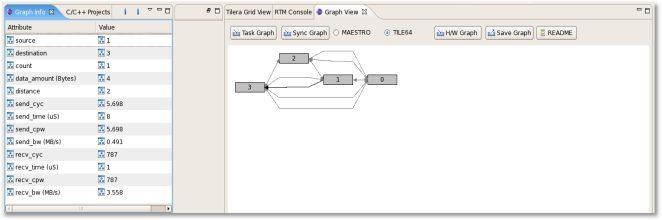
Message Passing Model Information.

In the message passing model, source and destination rank and tile location, transferred data amount, timestamp for each event are saved whenever an event happens and then collected by monitoring tile periodically according to the pre-defined interval. Using statistical results for each (source, destination) pair/process calculated by the RTM software server, the user can know that task dependency and the load, as shown in Figure 8.

**Figure 9.**
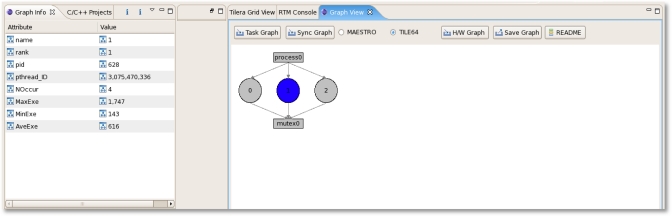
Shared Memory Model Information.

At the hardware level, Perfmon2 counter values were collected on each tile, transferred to the RTM hardware server and then provided as an XML file. The performance counters such as ONE, MP_BUNDLE_RETIRED, TLB_EXC, HIT, L2_HIT, MP_DATA_CACHE_STALL, MP_INSTRUCTION_CACHE_STALL, MISS_I, MISS_D_RD, and MISS_D_WR, were used for the hardware information. This information is collected by way of multiplexing on each tile and sent to the RTM hardware server. In Table 1 the characteristics of each performance counter are described:

In the shared memory model, the event name (Barrier/Mutex/Lock/Conditional events), the number of occurrences for each event, Max/Min/Ave time of each thread/process for each event, process/pthread ID, and the address of each event group are saved whenever an event happens and then collected by monitoring tile periodically according to the pre-defined interval. Using statistical results for each process/pthread/event, the user can know the synchronization information as shown in Figure 9. At the hardware level, it has no difference from the message passing model.

## 4. Implementation

The 64 cores are interconnected with mesh networks, while each processor executes at up to 866 MHz to achieve up to 443 billion operations per second. For supporting several libraries, we implemented MPI on the TILE64/TILEPro64/MAESTRO based on the MPI 1.2 specification. Perfmon2/PAPI was also ported on multi-core architecture by the research group of Professor Donald Young at The University of Maryland at College Park in collaboration with the USC/ISI.

**Figure 10.**
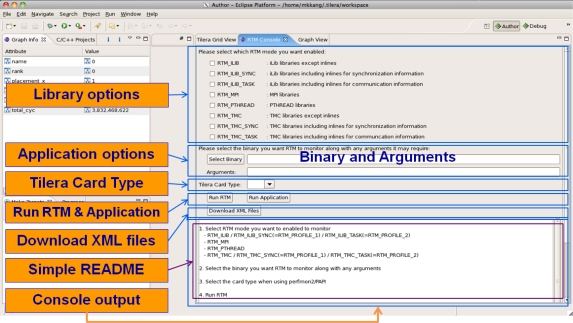
RTM Console.

**Figure 11.**
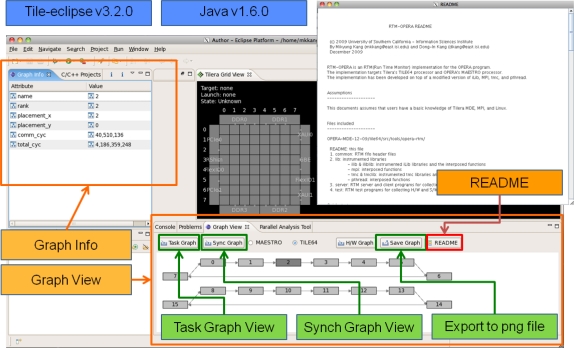
RTM Graph View and Graph Info.

Figures 10 and 11 depict the RTM eclipse plug-in which can be used for analyzing the periodic hardware and software results on TILE64/TILEPro64 or MAESTRO. After the user can select library options, application options, and multi-core environments and then run RTM with applications using the RTM console plug-in. These tile commands can also be run as a script by a node controller on Eucalyptus. Once RTM is run, the collected information is saved, analyzed, generated to XML format, and provided to RTM Graph View and Info plug-in offline.

**Figure 12.**
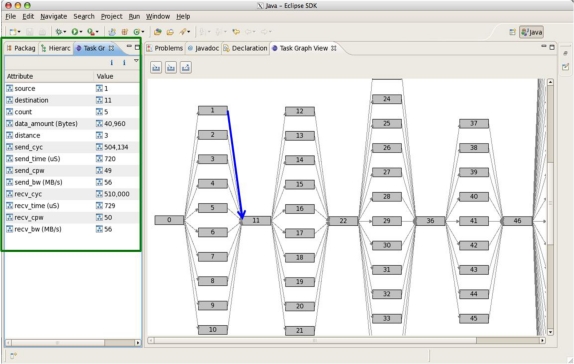
RTM Task Graph.

Figure 12 shows a snapshot of the task graph for the communication pattern. As we mentioned in the previous section, the graph view and the related information can be provided. We can know the event’s source, destination, total count, data amount, distance, sender cycles, time, Cycles Per Word (CPW), bandwidth, and receiver cycles, time, bandwidth.

**Figure 13.**
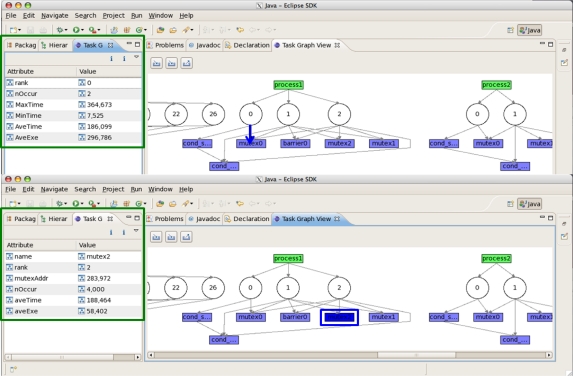
RTM Sync Graph.

Figure 13 shows a snapshot of the sync graph for the synchronization information. The first and second images show the result when the <link to the event/event> is selected. We can know the rank, the number of occurrences, maximum/minimum/average cycles, and the average execution time between each event.

The RTM hardware server gathers performance counter information and provides in a XML file. Using this XML file, user can know the current status of which tile is running.

This RTM has been already demonstrated on Multi-Core Architecture, and the multi-core architecture has been set for Cloud Computing, so the proposed monitoring can be run in the context of Cloud Computing. The overhead introduced by our performance assessment tools varies significantly with the specific tools and calls and the application. PAPI is a standard, portable API that provides an interface for performance counter hardware. PAPI provides a set of function calls that allow the programmer to control and read performance counters that are implemented in the hardware. The performance overhead of using PAPI is under control of the programmer, since calls to PAPI functions are manually inserted. Performance impact is typically low since the actual event counting is done in hardware. For example, a program may set up a performance counter to monitor cache misses at the beginning of a program, let the program execute normally for many seconds, and then read the results of the counter, in which case the performance overhead of PAPI would be negligible. A call to a PAPI function can vary depending on the function and the arguments but is comparable to a system call. The overhead introduced by PAPI for a specific usage can be analyzed by running the program with and without PAPI and using the get_cycle_count function call to measure the execution time.

The run-time monitor that USC/ISI has developed uses instrumented versions of common libraries to collect information at run-time about program behavior. The run-time monitor collects information about communication for message passing programs and about synchronization calls for shared memory programs. For iLib messages passing for small messages, overhead for performance monitoring is very large—roughly 10x the execution time of the base function call. This is expected since iLib is intended to provide access to the UDN with little overhead, and the performance monitoring code can be much more complex. For applications that send small messages frequently, this overhead would be unacceptable because the perturbation to the original program behavior would be enough that gather performance information on the instrumented program would be irrelevant. Programs with less frequent message passing would see less perturbation. For larger message sizes, overhead is much more reasonable. For example, for a message size of 64 K words (256 KB), overhead is less than 30%. The overhead introduced to pthread synchronization calls can also be significant. Instrumentation causes the cost of calls to lock and unlock functions to increase by a factor of 20. For programs that make frequent calls to available locks, this overhead would be unacceptable. However, for programs that are frequently waiting for locks (making the execution time of uninstrumented locks much longer), this overhead may be much more tolerable. As with all performance analysis techniques, the programmer must consider the affect of the measurements on program behavior and use the tools accordingly.

## 5. Conclusions

In this paper, we have designed a Run-Time Monitor which is a system software to monitor the characteristics of applications at run-time, analyze the collected information, and optimize resources for cloud computing. RTM monitors both application software through library instrumentation and underlying hardware through performance counter optimizing its computing configuration based on the analyzed data. For future work, we are planning to develop a dynamic run-time self-morphing software framework on multi-core systems. It is expected to provide a framework for an automated optimization of the software with minimal overhead on multi-core systems. After all, the key feature of our framework is that: (1) performance monitoring is detached from the applications to system-wide run-time manager, (2) the application has a range of morphing at run-time, (3) the run-time manager monitors the application’s performance and morphs the application at run-time for either better performance or adapting to a situation.

**Figure 15.**
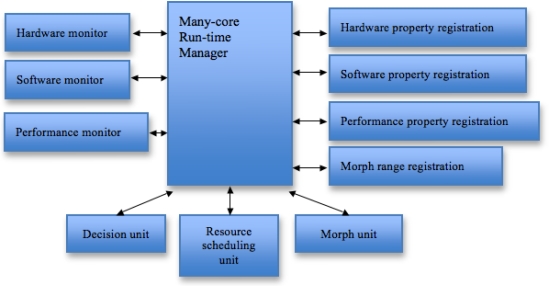
Overview of run-time dynamic self-adapting software framework.

An overview of a run-time dynamic self-adapting software framework is shown in Figure 15. An application has a range of self-morphing internally. RTM manages the system resources and decides resource allocation, scheduling, and morphing for the applications. RTM monitors the applications and morphs the applications when it is needed. An application informs RTM of its own characteristics that include the ranges of morphing and the properties of each morphing. RTM monitors applications using hardware monitor, software monitor, and performance monitor.

## Figures and Tables

**Figure 14.**
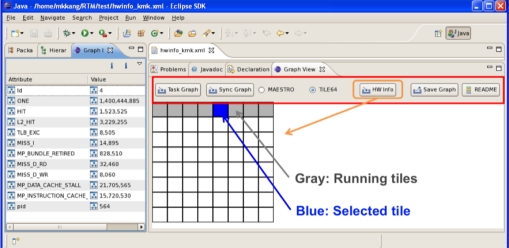
RTM Hardware Info Graph.

**Table 1.** Performance counter.

**Counter**	**Description**
ONE	Clock cycles
MP_BUNDLE_RETIRED	The event occurs when an instruction bundle is retired
TLB_EXC	The event occurs when the address of a data stream memory operation causes a Data TLB Exception including TLB Misses and protection violations
HIT	This event occurs when a load instruction hits in the L1 Data cache
L2_HIT	This event occurs when any cache access hits the L2 and includes MDN fills and Memory Fence operations locally or remotely issued
MP_DATA_CACHE_STALL	An event occurs every cycle that an instruction bundle is stalled on a data memory operation, except for the cycles when a replay trap is being performed. Instructions that depend on the result of a load and are fired speculatively cause a reply trap if the request misses the L1 data cache and thus are not counted. The wait is 4 if the consumer of the load immediately follows the load or 3 if there is a cycle between the load issue and the consumer issue. Multiple stall events may occur and be counted during the same cycle
MP_INSTRUCTION_CACHE_STALL	An event occurs every cycle that an instruction bundle is stalled on a instruction memory operation. Multiple stall event occur and be counted during the same cycle
MISS_I	The event occurs when an instruction stream read misses the L2 cache due to an L1 instruction cache miss
MISS_D_RD	The event occurs when a load request or instruction prefetch misses the L2 cache due to an L1 miss with the page cached locally or remotely
MISS_D_WR	The event occurs when a store request misses the L2 cache with the page cached locally or remotely
